# The effectiveness of naive optimization of the egress path for an active-shooter scenario

**DOI:** 10.1016/j.heliyon.2023.e13695

**Published:** 2023-02-13

**Authors:** Joseph Lavalle-Rivera, Aniirudh Ramesh, Laura M. Harris, Subhadeep Chakraborty

**Affiliations:** Mechanical, Aerospace and Biomedical Engineering Department, University of Tennessee, Knoxville, 1512 Middle Dr, Knoxville, TN 37996, USA

**Keywords:** Active-shooter scenario, Egress, Non-homogeneous semi-Markov decision process, Building emergency evacuation

## Abstract

There have been 130 mass shootings in the United States from 1982 to June, 2022 according to the Mother Jones database of active shooter events. In these critical scenarios, making the right decisions while evacuating can be the difference between life and death. However, emergency evacuation is intensely stressful, which along with lack of verifiable real-time information may lead to costly incorrect decisions. In this paper, we demonstrate the effectiveness of a non-homogeneous semi-Markov-Decision-Process (NHSMDP) based naive algorithm that relies on prior knowledge about the layout of a building and uses recurring updates of the shooter's location (based on automatic processing of images from a camera network) to provide an optimized egress plan for evacuees. While emergency evacuations due to fire and natural disasters are well researched, the novelty of this work is in the response to a threat that moves either purposefully or randomly through the building and in incorporating the ability for an evacuee to wait for danger to pass before beginning egress and during the process of evacuation. This ability to include sojourn times in the optimized scheme is due to the NHSMDP formulation and is a notable augmentation to the current state-of-the-art. We show that following this algorithm can reduce casualties by 56% and the time spent by evacuees in the shooter's line of sight by 52% compared to an intuitive natural response guided by expert advice.

## 1 Introduction

Emergency evacuations are a topic of great interest to researchers from many different backgrounds. Whether it is traffic evacuation in the event of disasters like earthquakes and hurricanes, analysis of human behavior during evacuation proceedings, or the movement of crowds within the context of an evacuation, the approaches to manage and optimize these evacuations are widely varied and thoroughly researched. The impetus for the quantity of the research conducted is directly related to the importance of ensuring safe and effective egress for all evacuees in each of these situations as they are usually characterized by great danger. Improvements in speed, effectiveness, and efficiency in all evacuation situations result in fewer casualties and the pursuit of situational improvements is sufficient rationale for such optimization. The interested reader can find a comprehensive literature review and mapping of the research conducted in the area of emergency evacuations in the survey paper by Liu et al. [18]. They identified three main knowledge groups within the literature: traffic evacuation, group behavior, and crowd evacuations. The methods, approaches, and rationale within these knowledge groups vary greatly as is expected in such an open problem. For example, Lin et al. focused on human behavior within crowds during building evacuations [17]. Others have focused on specific groups, like Duleb et al., who studied vulnerable populations that require additional preparation in order to effectively evacuate in a given situation [8]. Some researchers like Abdulhalim et al. have identified very specific and unique populations, like deaf and hard of hearing children, that require special consideration when determining evacuation proceedings [2]. Mirahadi and McCabe developed a fire evacuation model based upon Dijkstra's shortest path algorithm [22], but they, like most of the available research on evacuation planning and execution, still expect to route all personnel to safety as quickly as possible, but do not consider the option of “waiting out” the danger. It is quite reasonable to assume that in most usual hazards, such as fire, earthquake, etc. fastest evacuation is the safest evacuation.

However, the focus of this paper is on evacuation during an active shooting incident, an unfortunately relevant and recurring emergency that necessitates us to diverge from traditional solutions, since unlike natural disasters, the threat from shooters is localized around the shooter, very mobile and transitory. Therefore, hiding before and during evacuation (by temporarily darting in to safer rooms) are options worthy of exploring. While the literature on egress is vast and a good source of inspiration, the unique nature of active shooting events poses unique algorithmic challenges that are discussed in this paper.

While emergency evacuations are extremely stressful events, they are even more so during an active shooter incident and the confusion and panic along with lack of verifiable information can lead to sub-optimal, short-sighted decisions. The Mother Jones Mass Shootings Database classifies mass shootings as events where a shooter with firearms opens fire on a large number of people, killing at least four victims. According to a study, public mass shootings have occurred 128 times between 1982 to 2022 in the United States [9]. Multiple studies suggest that the United States has had the highest number of mass shootings in the world [21], [26]. More than half of these shootings occur either in schools or workplaces [10]. In these critical scenarios, evacuees have to make key decisions such as whether to run or hide, or when and where to hide, or which direction to run, or whether to try and stop the shooter, etc. Getting these critical decisions right can mean the difference between life and death. The Department of Homeland Security advises evacuees in these scenarios to exercise the “Run-Hide-Fight” or “Avoid-Deny-Defend” protocols as a general strategy. The protocol advises evacuees to run and escape if possible as the first preference. If an exit is not accessible, the evacuees are asked to hide, stay silent, and barricade the rooms they are in. If neither “Run” nor “Hide” is an option, the evacuees are advised to aggressively fight the shooter as the absolute last resort. However, without specific information about the shooter's location, and without guidance while escaping the building, this generalized “Run-Hide-Fight” protocol may not be effective, or worse, it can be misleading. Without guidance in the escape plan, the evacuees may run toward the shooter rather than away, or come out of their barricaded room at the wrong time. In fact, this happened during the Parkland School Shooting in 2018, where a flood of people ran straight towards the direction of the shooter [6]. Further, these situations are, in most cases, so short in duration that when the first responders arrive, often within three to five minutes, the vast majority of the damage is already done, making the initial actions taken by people involved that much more critical.

A guidance system that tracks the dynamic location of the shooter in the building and communicates the safest egress plan to the evacuees based on this information has the potential to save lives during these unfortunate events. In this paper we exclusively focus on the routing strategies of the evacuees to maximize safety under a variety of conditions and situations, with the assumption that in any implementation, this algorithm will be paired with a camera-network based automatic shooter-identification and tracking system. We make no specific claims or impose constraints on the shooter-identification system, rather treat the camera locations and identification rates as independent parameters which affect the effectiveness and viability of our planning routine.

The interactions between the shooter and evacuees can be modeled in various ways – it may be conceived of as a non-cooperative game between competing players [31], as a pursuit-evasion problem [30] or as a graph search problem such as in the game of cops and robbers [25]. In this paper, we choose to decouple the shooter's decisions from the evacuees' activities, in the sense that the shooter pursues his own hidden agenda that we do not control nor have any knowledge of. This assumption is partly driven by the need to frame the egress route suggestion problem in an optimization framework, but also from practical considerations, since such motives are most often impossible to know or model a priori.

In this framework, we assume that the shooter's movement dynamics is described by a semi-Markov chain (SMC) with a discrete state space [11]. An SMC is a generalization of a traditional Markov chain with abstract sojourn time distributions. The evacuees' movements on the other hand, are modeled as a non-homogeneous semi-Markov system (NHSMS). This grants the model the capacity to incorporate both arbitrary waiting times at each state, but also an evolving transition probability which is based on the shooter's movements. For both shooter and evacuees, the discrete states of the underlying MDP are mapped into rooms, sections of hallways, stairways, and exits while the routes from one node to another are represented by edges. Since most analytical results in SMCs such as probabilities of occupancy, first passage times and duration of dwelling in a particular state are all obtained as distribution over an arbitrary interval of time, we take advantage of the short duration of the event (at most 5 minutes) to create an expanded state space by incorporating discretized time into the state definition. A finite horizon value iteration with non-homogeneous transition probabilities is then used to compute the optimal egress policy for all possible locations of the shooter and evacuees in the graph.

For the remainder of this paper, we first give a summary of current related literature and other works in section 2, describe the specifics of our NHSMS formulation in section 3, define the simulation environment used to evaluate the performance of both our ASTERS routing algorithm and a “Naturalistic Response” algorithm in section 4, discuss and compare the results from simulation in section 5, and, finally, identify future adaptions to the routing algorithm in section 6.

## 2 Literature survey

Emergency evacuations can be caused by a multitude of reasons including, but not limited to: earthquakes, hurricanes, floods, fires, active-shooter scenarios, etc. Given that time is critical in these evacuations, it is imperative that the evacuees choose an optimal route of escape as quickly as possible. Optimization of the routes taken by the evacuees, contingent upon their safety and a few constraints, can potentially save lives by maximizing the number of evacuees escaping safety.

Optimization of egress paths is a complex task as it not only is constrained by the physical geometry of the building, but also the capacity of different paths, dynamic-moving threats, etc. It should also account for human behaviors and factors such as herding mentality of people, panic, opinion propagation, misinformation, leader-follower dynamics, etc.

Hossam Abdelgawad and Baher Abdulhai completed a thorough review of the emergency evacuation literature in regards to transportation and, in particular, framing the problem as a network design problem [1]. They highlighted the benefits and shortfalls of using simulation as a method for testing and optimizing proposed solutions to emergency evacuations while also compiling proposed policies designed to increase the efficiency of said evacuations.

Thomas Kisko and Richard Francis together published their work on EVACNET+ [16] in 1985 in which they described an algorithm to determine optimal building evacuation plans. This was paired with a user-friendly interface that lets users model the building by representing the halls, rooms, etc. as nodes and passageways and corridors between them as edges. Then, the program outputs the optimal escape plan for that building floor plan to the user. This is done by using a capacitated network flow trans-shipment algorithm, which is a graph-theoretic approach to solving the network flow optimization problem. This algorithm is most suited for emergency evacuations caused due to fires.

Qingsong Lu and Shashi Shekar worked developing a capacity-constraint based evacuation algorithm [19]. They modeled capacity as a time series and used a capacity-constrained heuristic algorithm to determine the evacuation plan. They proposed an algorithm called Multiple-Route Capacity Constrained Planner (MRCCP), which produces near-optimal results but is significantly computationally less complex.

Farid Mirahadi and Brenda Y. McCabe focused on the evacuation of buildings in the event of a fire and proposed a real-time safest path based upon Dijkstra's shortest path algorithm [22]. They developed a risk factor for building compartments based upon proximity to the fire and potential blockages and produced routes that conformed to the Active Dynamic Signage System. The building is monitored in real time and the planned routes are adjusted as the event develops and their algorithm was shown to enhance the safety of evacuees.

Luh et al. in their work used a network-flow based novel stochastic dynamic programming to maximize the flow of people through the exits, given many other constraints [20]. They present a simulation of their algorithm which demonstrates rapid evacuation that avoids bottlenecking. AR Srinivasan discusses how decisions and opinions of evacuees can influence the entire group's behavior during these emergency evacuation scenarios [28].

Yin et al. approached the evacuation of dense, urban areas using agent-based modeling. They used phone location data to develop baseline evacuation plans based upon typical population densities [32]. They then used their offline database to accelerate the real-time adjustment of plans drastically decreasing the computation time required for real time development and slightly improving the performance.

Haghpanah et al. explored the evacuations of hospital, taking into account the different mobility disabilities of patients and requirements for distancing due to infectious diseases [13]. They introduced a non-ICU patient classification framework that identified the various mobility factors characterized by the patients conditions. They showed that guidance from a larger nursing team can greatly improve evacuation time while also demonstrating that a COVID-19 only exit much less effective.

Chou et al. approached building fire disasters from the aspect of routing the firefighters to appropriate locations to best assist evacuees [5]. The authors combined current firefighting equipment and procedures as well as Bluetooth technology, mobile network locations, communication systems, and signage to best direct the movement of evacuees and firefighters alike in a given indoor building fire. The proposed approach improved upon the number of casualties and the resources required to address the situation. This is potentially helpful in future work in active shooting evacuation research that will include routing of first responders to the threats location when they arrive upon the scene.

Balboa et al. used information provided by hazard detection systems, such smoke detectors identifying a fire's location, to calculate optimal routes that were the “fastest” (shortest according to Dijkstra's) and “safest” (maximized distance between the evacuation route and the hazard) within real-time simulations to guide evacuees [4]. They tested their approach in a multi-enclosure building with actual participants using a dynamic signage approach for guidance and demonstrated the effectiveness of real-time guidance for the evacuation of a building. They identified improvements in evacuee movement speed and required evacuation time lending credence to their hypothesis of the increase in performance based on intelligent dynamic signage. The use of dynamic signage and its effectiveness is promising as any real-time routing for an active shooter situation would need signage that does more than simply point in a direction.

Abusalama et al. examined a capacity constrained approach to routing and evacuation planning [3]. The authors developed a Dynamic Real-Time Capacity Constrained Routing algorithm that modeled capacities based upon time series to address the Emergency Route Planning problem. They demonstrated an improvement on both computation required and time for evacuation. As indicated in most of this literature, this work does not account for the mobile threat to the evacuees, nor the ability for an evacuee to hide from said threat.

Despite the literature for emergency evacuation planning in general being vast and well documented, works on egress solutions for active-shooter scenarios are very scant. This may be partially attributed to the complexity of the problem. Such events last very few minutes and are highly stochastic. Moreover, due to the dynamic nature of the shooter, the evacuation plan has to be dynamic as well. Gunn et al. modeled an optimal egress plan for active shooter scenarios [12]. They developed a novel divide-and-conquer approach to divide evacuees into groups and used dynamic stochastic programming for finding the optimal paths for these groups. However, their work did not take into account several important factors like dynamic shooter movement, the shooter's line of sight, human factors, capacity constraints, etc. To our knowledge, no work has been done so far that solves an egress plan for active-shooter scenario that dynamically updates as the location of the shooter changes.

## 3 Methodology

We model the shooter's movement by a semi-Markov chain ${\left\{X_{t}\right\}}_{t\geq 1}$ with state space $S^{s}=N_{1}^{s},N_{2}^{s},...,N_{n}^{s}$ corresponding to nodes in the building layout. Here *n* is the total number of nodes and the superscript *s* signifies that this state space corresponds to the shooter's movement. Later on, we will define $S^{e}=N_{1}^{e},N_{2}^{e},...,N_{n}^{e}$ for the evacuee's state space which physically overlaps with the shooter's space, but conceptually part of a separate NHSMDP setup.

The successive states/nodes chosen by the shooter to visit are defined by the transition probability matrix and the sojourn time in each node, conditioned on the current and the next state to be transitioned into. Thus, during the transition times, the process is equivalent to an embedded Markov process. Let transition probabilities $p_{N_{i}^{s}\to N_{j}^{s}}(t)$ be the probability of the shooter who entered state $N_{i}^{s}$ during the last transition at time *t* to transition to state $N_{j}^{s}$ in the next transition. The transition probabilities should satisfy the same equations of a Markovian process, that is, $p_{N_{i}^{s}\to N_{j}^{s}}\geq 0,\forall N_{i}^{s},N_{j}^{s}\in S$ and $\Sigma_{j=1}^{n}p_{N_{i}^{s}\to N_{j}^{s}}=1,\forall N_{i}^{s}\in S$.

When the shooter enters node $N_{i}^{s}$ at time *t*, we assume that this node determines the next transition to node $N_{j}^{s}$, which occurs according to the transition probabilities. However, before making the transition from state $N_{i}^{s}$ to state $N_{j}^{s}$ and after the next state $N_{j}^{s}$ is selected, the chain holds in state $N_{i}^{s}$ for time $t_{ij}$. The sojourn time $t_{ij}$ is a positive random variable with density function $h_{ij}()$, which is called the function of sojourn time to transition from state $N_{i}^{s}$ to state $N_{j}^{s}$. Thus, $P(t_{ij}=m)=h_{ij}(m)$, for m=1,2,.., and $N_{i}^{s},N_{j}^{s}\in S$. We assume that the mean values of the distributions of sojourn times are finite.

In the context of the shooter's movement, various types of motivations and personalities can be modeled with judicious choice of the sojourn time distribution and the transition probabilities. Consultation with topical experts has suggested that there are three primary personality profiles that characterize a shooter [24]

• either a shooter tries to maximize damage and thus seeks out the most densely populated areas in the building,
• or a shooter is following a personal motive and seeks out a specific target,
• or a shooter is carrying out an unplanned act of violence.

**Figure 1:**
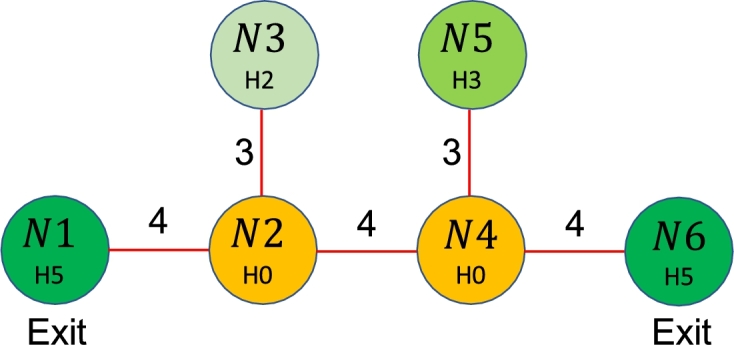
Toy problem used to demonstrate the dynamics of transitions.

Since it is almost impossible to predict beforehand the motive prompting a particular type of shooter, and moreover, the cost of an erroneous determination can be catastrophic, in this paper we assume a memoryless uniform probability discrete choice model, which can be explained with the help of a simplified example, shown in Fig. 1.

We start with the assumption that we know the location, i.e. the state of the shooter at t=0. The memory-less uniform probability discrete choice model dictates that at any instant, the shooter can choose to stay in the current node for the duration of the discretized decision cycle, or start moving to any of the adjacent nodes based on the building layout. Without knowing or trying to predict the shooters actions, we assumed the worst-case scenario for planning purposes that the shooter could go in any direction with equal probability. Again, using the toy problem setup, a shooter in $N_{4}^{s}$ at t=0 would choose to move to the adjacent nodes with probabilities $P(N_{2}^{s})=P(N_{4}^{s})=P(N_{5}^{s})=P(N_{6}^{s})=0.25$. With this assumption of memoryless uniform choice probability, the cascading probabilities of the shooter's positions at the various nodes and edges can be computed. Table 1 below demonstrates this. It may be noted that at t=1 $P(N_{4}^{s})=0.25$ whereas the probability the shooter is on an edge moving to the node of choice is 0.25 as well, i.e. $P(E_{2\to 4}^{s})=P(E_{4\to 5}^{s})=P(E_{4\to 6}^{s})=0.25$. Based on the travel time between nodes, the probabilities of reaching nodes $N_{2}^{s}$, $N_{6}^{s}$ and $N_{5}^{s}$ spike at 4,4 and 3 seconds respectively. This sequence of unbiased discrete choice between the accessible nodes, travel time and sojourn time at the nodes is cascaded until we can compute the probabilities of the shooter being in any of the accessible rooms in the building at any subsequent times. This propagation of probability continues throughout the 300 seconds which is set as an upper bound of the duration of these events based on expert advice.

**Table 1:** Probable location of shooter at time = *t*.

Time in seconds	0	1	2	3	4	5	6	7
*N*_1_	0.00	0.00	0.00	0.00	0.00	0.00	0.00	0.00
*N*_2_	0.00	0.00	0.00	0.00	0.25	0.13	0.05	0.02
*N*_3_	0.00	0.00	0.00	0.00	0.00	0.00	0.00	0.06
*N*_4_	1.00	0.25	0.06	0.02	0.00	0.00	0.13	0.13
*N*_5_	0.00	0.00	0.00	0.25	0.19	0.11	0.06	0.03
*N*_6_	0.00	0.00	0.00	0.00	0.25	0.19	0.11	0.06
*E*_1→2_	0.00	0.00	0.00	0.00	0.00	0.06	0.09	0.11
*E*_2→3_	0.00	0.00	0.00	0.00	0.00	0.06	0.09	0.04
*E*_2→4_	0.00	0.25	0.31	0.33	0.09	0.09	0.10	0.14
*E*_4→5_	0.00	0.25	0.31	0.08	0.15	0.22	0.15	0.12
*E*_4→6_	0.00	0.25	0.31	0.33	0.08	0.15	0.22	0.31

A limitation of this shooter-movement model is that, we assume that the shooter does not turn back or change his decision while halfway to a target node, i.e. each sequential target choice is made only after each node is reached, but not in the links connecting these nodes. Moreover, the memoryless choice dynamics may be improved, by more realistically modeling the sojourn times to follow a specific distribution. However, this model does provide a robust baseline for evaluating the performance of the algorithm for routing the evacuees away from the shooter. The evacuation optimization algorithm is discussed next.

### 3.1 Evacuation algorithm

The algorithm used to determine the routing of the evacuees follows a practical implementation of the non-homogeneous semi-Markov decision process (NHSMDP) optimized with finite-horizon value iterations. A Markov Decision Process formulation is defined by the 4-tuple $\left(S,A,P_{a},R_{a}\right)$ where *S* is a set of possible states, *A* is a set of possible actions that can be taken from these states, $P_{a}(s,s^{\prime })=P(s_{t+1}=s^{\prime }|s_{t}=s,a_{t}=a)$ is the probability that action *a* in state *s* at time *t* will lead to state $s^{\prime }$ at time t+1, and $R_{a}(s,s^{\prime })$ is the expected immediate reward received after transitioning from state *s* to state $s^{\prime }$ due to action *a*. In contrast to standard MDPs, the NHSMDP differs in two main aspects, the non-stationary transition probabilities and the idea of sojourn times at various nodes. These differences and their treatments are explained next in the context of an egress plan.

#### 3.1.1 State

In the context of an egress plan, the state is a tuple defined by $S^{e}=\{N^{e},t\}$, where $N^{e}$ are the nodes that the evacuees can occupy, and t∈T is quantized time, elapsed since the shooter was last spotted by a camera. By folding time into the state definition allows us to include plans for waiting in a state, but exponentially increases the size of the state space. However, statistics and expert consultations suggest that most active shooter situations culminate within 5 minutes or 300 seconds. Quantizing time at 10 seconds intervals (any higher resolution would be irrelevant, since the slow speed of human movement dynamics would not allow any appreciable changes at finer time scales) yields |T|=30 time steps. To put that in context, for a large building with 100 nodes (say), for each shooter position, the optimization space would be $|N^{e}|\times |A^{e}|\times |T|=100\times 100\times 30=3000$, a level of complexity that can be handled by modern computers, especially since the optimization is completed off-line and stored as a policy.

#### 3.1.2 Action

In our problem, the evacuees' actions $A=\{M_{S,T}^{e}\}$ represent the decision to move from a certain source node ($N_{S}^{e}$) toward a certain target node ($N_{T}^{e}$) at each discretized time step until they reach safety. Based on the layout of the building, the action can either be to stay in their current node until the next decision cycle, and then choose another action, or they can begin moving towards any adjacent node in the graph network starting from their current node location.

#### 3.1.3 Transition probability

**Figure 2:**
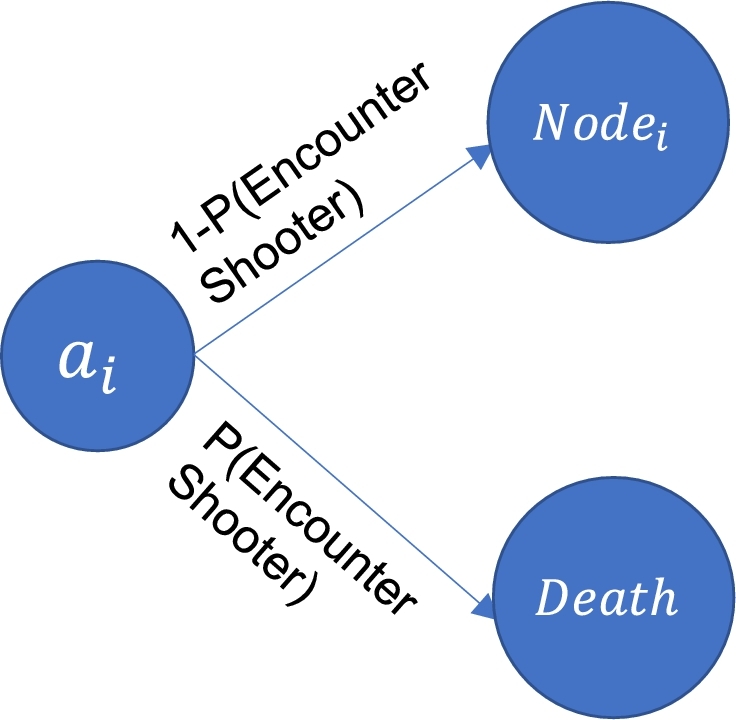
Probabilities associated with an action.

The probabilities associated with successfully executing the chosen action are calculated based on the shooter's movement model. It is assumed that the evacuees' probability of transitioning to their target node within a certain time is entirely dependent on them not encountering the shooter en route and is portrayed in Fig. 2. This may be calculated by inputting elements from Table 1 into Equation (1) for each step in the evacuees path from source to the target node.

(1) $$P(N_{S}^{e},N_{T}^{e})=1-max(P(N_{i}^{s}),P(E_{i,j}^{s})),\;\forall N_{i}⋃E_{i,j}$$

Unlike traditional MDPs, the transition probability is an evolving function of time depending on the shooter's last known location and his possible movement choices. This non-stationary transition matrix makes this an NHSMDP.

In the context of the toy problem, Nodes $N_{1}$ and $N_{6}$ are exits and the values on the edges are travel times between nodes. Each node also has a hardness attribute that will be addressed in the reward structure discussion. We conjecture that an evacuee, without taking into account willingness to comply, the uncertainty associated with guiding young children, etc. can successfully stay in their current node or move to another node with probability 1.00 unless there is an interaction with the shooter.

Here interaction is defined as either being in the same node as the shooter or being in his line of sight defined by the layout geometry, for example, the line of sight for a hallway node would include all nodes along the length of that hallway, while the line of sight from inside a room does not extend beyond the room itself. Table 2 demonstrates this modification which in essence changes the meaning of the probability to be the likelihood that the evacuee will be in the line of sight of the shooter.

**Table 2:** Probability of harm by shooter at time = *t*.

Time in seconds	0	1	2	3	4	5	6	7
Node 1	0.00	0.00	0.00	0.00	0.25	0.13	0.09	0.11
Node 2	1.00	0.25	0.31	0.33	0.25	0.13	0.13	0.14
Node 3	0.00	0.00	0.00	0.00	0.00	0.06	0.09	0.06
Node 4	1.00	0.25	0.31	0.33	0.25	0.22	0.22	0.31
Node 5	0.00	0.25	0.31	0.25	0.19	0.22	0.15	0.12
Node 6	1.00	0.25	0.31	0.33	0.25	0.19	0.22	0.31

#### 3.1.4 Reward structure

The final initialization of the NHSMDP is the reward structure that determines the benefit or detriment that goes along with executing a given action from a specified state. Our reward structure is based upon the hardness of the node and the time to reach the nearest exit from that node. We consider the hardness of the node as a metric of how much protection the node can offer against a potential shooting, i.e. an open hallway node has a hardness score of 0 whereas a corner room with no windows will have a higher hardness score. An exit would have the largest hardness score since it provides the chance to escape away from the building. In a practical implementation, the hardness would be based upon the type of door, presence of windows, and the perceived building materials used in the construction of the room. The times to the nearest exit for each node were calculated based upon Dijkstra's shortest path algorithm [7] using travel time as the weight. Incorporation of this distance-from-exit metric in the reward incentivizes the algorithm to prioritize routes that lead towards one of the exits rather than move the evacuees deeper into the building in search of safer rooms. The reward equation follows:

$$R(N_{S}^{e},N_{T}^{e})=\left\{ \begin{array}{c} \alpha \times \frac{h(N_{T})-h(N_{S})}{h_{max}-h_{min}}+(1-\alpha )\times \frac{\tau (N_{S})-\tau (N_{T})}{\tau_{max}-\tau_{min}}\\ 10\;\;\;\;\text{if action leads to an exit}\\ -10\;\text{if action results in an interaction} \end{array}\right.$$

where $h_{S}$ and $h_{T}$ represent the hardness of the source and target nodes respectively, while *τ* represents the shortest time to reach the nearest exit. *α* is a tunable factor that can be engineered to more heavily weigh either the hardness metric or the distance-from-exit metric in the reward function. A small parameter sensitivity study was performed to find an optimal value of α=0.75.

The overarching idea behind this reward function design is that if the evacuee moves from a “softer” node to a “harder” one, they gain a positive reward by virtue of moving to a safer node and vice-versa. The opposite is true of the time to exit for each node. An action that leads to an exit receives a reward of 10 and an action that leads to an encounter with the shooter receives a reward of −10. The rewards for reaching an exit node or the shooter-occupied node are constant and each represent a terminal state.

#### 3.1.5 Value iteration

With the framework of our problem set, we determined the optimal actions in each state using value iteration [29]. For each location of the shooter, the finite horizon optimization problem was over the defined NHSMDP. The values for the states are calculated according to the equation

$$V^{N_{s}}(N_{S}^{e},t)=max_{a}P(N_{S}^{e},N_{T}^{e})\times R(N_{S}^{e},N_{T}^{e})+P(N_{S}^{e},N_{T}^{e})\times (\gamma V(N_{T}^{e},t+\tau (S\to T)))$$

where $N_{s}$ is the current known node of the shooter, $N_{S}^{e}$ is the current (Source) node of the evacuee, $N_{T}^{e}$ is the destination node, $P(N_{S}^{e},N_{T}^{e})$ is the probability of the evacuee reaching the target node at time t+τ(S→T) starting from the source node at time *t*, where τ(S→T) is the travel time between the source and target nodes. $R(N_{S}^{e},N_{T}^{e})$ is the reward for moving from source to target, *γ* is the discount factor (0.75 in our case). The expected reward for every action for every state is compared to the current value of the state. If it is better, the value is updated. If it is not better, the value remains the same and the algorithm moves onto the next action. This process is iterated through each state-action pair until the largest change is smaller than ϵ=0.0001 or a maximum number of iterations (1000 in our case) is reached. Once the value iteration has converged, the value iteration process is iterated through one more time to record the optimal action in each state. The optimal action becomes the routing path determined by the current state of shooter node, evacuee node, and current time. An example routing plan for an evacuee located in $N_{5}$ when the shooter is located in $N_{4}$ would be $\left(N_{5},N_{5},N_{5},N_{5},N_{5},N_{5},N_{4},N_{6}\right)$. This plan demonstrates how the evacuee should wait in $N_{5}$ for 6 cycles that is 60 seconds before moving to $N_{4}$. This process is completed for each of the possible starting shooter locations. The result is a look-up table with an entry for every combination of shooter position, evacuee position, and time elapsed that will direct the evacuees to safety in the simulation environment.

### 3.2 A broader perspective on the choice of NHSMDP as a modeling framework

Due to the specific nature of the challenges related to an active shooter scenario, two aspects of the optimization objective made it uniquely different from most methods found in evacuation and egress literature:

1 A complex mobile threat that is dangerous within a certain vicinity of the shooter and consequently is transient; and
2 The need to maintain the ability of an evacuee to wait, hide, and proceed with evacuation.

An optimization structure built around NHSMDP addresses both of these challenges. From a language theoretic point of view, a semi-Markov chain and a deterministic finite automata (DFA) are identical, which can be informally argued as follows: Let the list of each node visited by the evacuee be expressed as a string *w* and the collection of all such possible strings denoted by the language L={w}. The string *w* can always be broken into three strings w=xyz; such that, $xy^{k}z\;\forall k\geq 0$ is also in L. The *k* repetitions of *y* here imply that the evacuee stay hidden in Node *y* for *k* decision epochs. This is the basis of the Pumping Lemma [14] that proves that L is a regular language and hence can be expressed by a deterministic finite state automata. It is crucial to note that this is only valid as long as there is no restriction on the number of epochs an evacuee can stay in one node. If additional restrictions are applied on the path taken, in some cases, the language L might cease to be a regular language and the NHSMDP formulation would not be appropriate.

Moreover, since we are not merely interested in expressing possible routes, but maximize the probabilistic reward, we have to account for the change in probability as a cascading function of the shooter's movement in time. The simple act of quantifying the product space of time and nodes as the state space of our NHSMDP $\left(S^{e}=\{N^{e}\times t\}\right)$ gives the formulation sufficient expressive power to allow estimation of cumulative gains from waiting in the same node for multiple epochs and then venturing out towards safety. For example, in Fig. 1, given a state tuple of $S=(N_{4}^{e},N_{1}^{s},0)$, our algorithm would guide the evacuee to $N_{5}^{e}$ as moving to the exit keeps them in the shooter's line of sight for longer. If the shooter then moves to $N_{3}^{s}$, our evacuee could then potentially make their move to the exit. Most routing research currently identifies shortest or fastest routes, not necessarily the safest.

The nature of the problem along with the short duration which makes it feasible to work with a time expanded state space creates an unique opportunity to use a model based optimization. In comparison to other potential methods in machine learning such as Deep Reinforcement Learning, this provides a logical explainable methodology with guaranteed optimization results.

## 4 Experimental design

### 4.1 Simulation environment

In order to evaluate the performance of our algorithm, we used the package “Simpy” [23], a process-based, discrete event simulation framework within Python, to create an environment where a shooter could act within a building environment and allow multiple evacuees to respond according to the routing produced by the routing algorithm.

Once the simulation was completed, performance metrics such as number of casualties, escapes, the amount of time spent within the shooter's line of sight, and the number of times an evacuee's routing plan was changed, were recorded. The time in the shooter's line of sight was evaluated by comparing each evacuee's current position to the shooter's position with respect to the specific layout of the building at every point of simulation time.

The deterministic metric for casualties was an extension of the line-of-sight evaluation. An evacuee was considered to be a casualty if the evacuee's node at any time was less than 4 edges away (based on Djakstra's shortest path algorithm) from the shooter's node while being in the shooter's node's line of sight. The length of 4 is based upon the generalized accuracy and lethality of a hand gun according to law enforcement professionals. An evacuee who reaches an exit node before being caught by the shooter was considered to have escaped. Each of these metrics was collected for every second of time up to the 300 second threshold or until every evacuee had either escaped or been caught.

### 4.2 Shooter actions

The actions of the shooter were partially predetermined and randomized based upon the layout of the simulated building. At the beginning of the simulation, the shooter was initialized, which means the shooter spawned in a predetermined node with an attribute of a target node (both the spawn node and the target node are set by the parameters of the simulation and will be discussed later). Upon spawning, the shooter was programmed to determine the shortest path to its target node and to begin moving along that path (since most active shooter events are pre-planned, it is likely the shooter would know the fastest way to get to the desired location) [24]. Whenever the shooter reached the first way-point node along his route, it was removed from the planned path. This continued until the shooter reached the target node. Upon reaching the target node, the shooter was programmed to stay in the node for 5 seconds, after which, a new target node was randomly selected from the remaining unvisited rooms in the building, with closer rooms having a higher probability of being selected, and the shooter would begin moving in that direction. This process of the shooter randomly moving from node to node continued until either all evacuees had escaped/died or the 300 second threshold had been reached.

### 4.3 Evacuee actions

The actions of the evacuees are based on the optimum route determined based upon their location with respect to the shooter's. For the purposes of determining the efficacy of the algorithm without considerations for compliance or other human-factors, we assumed that the evacuees would all perform the routing instructions without hesitation. In the same fashion as the shooter, the evacuees would be provided a complete path to an exit. That path may require the evacuee to start moving immediately, hold in a room for one or multiple decision cycles before moving, based upon the proximity of the shooter. Also, like the shooter, the evacuees do not make new decisions during the travel to the target node. Upon reaching the next node, the evacuee compares their current position to that of the shooter and either continues on the previous planned route, or changes the route because the state of the system has changed.

### 4.4 “Naturalistic response”

We are going to refer to a set of simple rules that encode the egress logic behind the “run-hide-fight protocol” as the Naturalistic Response (NR) and use NR as the basis for comparison with the performance of the optimized routing algorithm. For a fair comparison, the NR algorithm would use the exact same information about the shooter's position as well as knowledge of the building layout and the ability to determine the shortest paths to all exits. The NR algorithm can be described as a set of rules as follows:

• At every decision update point, if an evacuee is in a hallway, he/she will either start running toward an exit or towards the nearest room based upon the proximity of the shooter.
• If the evacuee is in a room and if the shooter is within a certain proximity threshold path length of the evacuee's node, they will hide in the room.
• When the shooter is farther away than the threshold path length, the evacuee will begin moving toward the nearest exit.

For our simulation, we examined and compared the casualties and LOS times by varying the proximity thresholds from 1 to 8, named $NR_{1}$, $NR_{2}$, etc. for ease of reference. For example, for plan $NR_{8}$, an evacuee will hide in a room as long as the shooter is within a path length of 8 nodes and begin moving as soon as the shooter is farther away.

### 4.5 Graph environments

**Figure 3:**
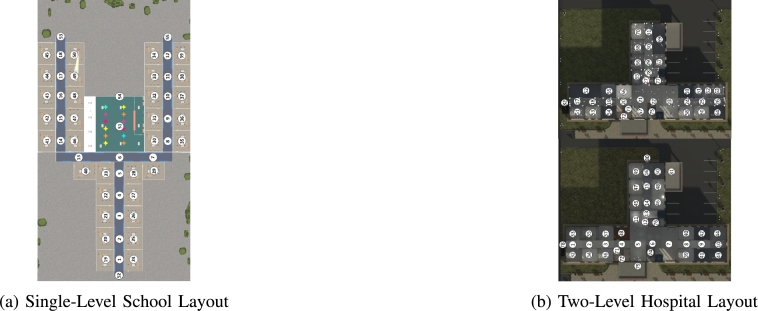
Two different graphical layouts for simulation comparison.

We simulated the evacuation procedures in two different buildings: a single-level, three-wing school (Fig. 3a) and a two-level hospital (Fig. 3b). The algorithm, because of its reliance on the estimation of probabilities of evacuees encountering the shooter, needs to know some layout specific parameters, such as the distance between nodes, relative safety of nodes which we call hardness, and the line of sight from a given node along with the travel time between nodes. To get a realistic estimate of travel times between nodes, in the absence of real data, we used simulations created in Unity and Unreal Engine to determine the time to go from one node to another. Because these gaming engines utilize physics-based models of movement – walking, running, and even model physical conflicts caused by obstructions, proximity and bottlenecks, we believe that the simulated travel times are reasonable estimates of actual movements. We performed simulations between each node pair and averaged travel time over 10 iterations.

The safety of a node, or hardness, was a subjective value that is more meaningful in the context of real-world implementation. For the purpose of our algorithm, all nodes in hallways or stairways were determined to have a hardness of *0* which implied that these spaces offered no protection whether from cover or concealment. An exit was determined to have the highest hardness as it is projected as offering the most safety in the form of escape. For out two graphs, both exits were given hardness values of *8* with interior rooms given hardness values in between the minimum and the maximum. The values for the rooms were subjective, but meant to reflect the level of relative protection offered, such as, a room with a single entrance and a windowless metal door that can be barricaded had a higher value than a room with multiple entrances and windows in the door. Further, in order to ensure the subjectivity of the values didn't overly affect the routing decisions, the reward function normalized the values on a scale of *0-1* as discussed in section 3.1.4. The line-of-sight array was simply a list if nodes that could be seen from a given node and was manually determined based upon the graph network and building layout.

We experimented with the movement speed of the evacuees relative to the speed of the shooter. We simulated different types of evacuees by allowing the evacuees to move at 50% (young children), 75% (middle school to high school), and 100% (college and adult) of the shooters speed. We also experimented with the distribution of the evacuees: either the evacuees were uniformly distributed only in the rooms, or they occupied both rooms and hallways. The final parameter we examined, which was common to both buildings, was the rate at which the evacuees were updated about the shooter's position. The position updates based upon time were tested at values of 30, 20, 10, and 1 second intervals with the 1 second update interval simulating full and continuous knowledge of the shooter's location.

The single-level school can be seen in Fig. 3a. The hallways are indicated by nodes 1 through 18, the rooms are indicated by nodes 19 to 50, $N_{51}$ is the cafeteria, and the exits are indicated by nodes 52, 53, 54, 55. The two-level hospital can also be seen in the Fig. 3b. Similarly to the school, nodes 1 through 31 are the hallways, nodes 32 through 70 are the rooms, nodes 71 through 77 are the stairways, and nodes 78 through 82 are the exits. The remaining parameters, which contained multiple values and were distinct and specific to the school and hospital, were the spawn locations of the shooter, the shooter's initial target room, and camera locations. For the sake of clarity, the details of each of these parameters, will be discussed in more detail in the results section. The shooter's initial target rooms ($N_{26},\;N_{36},\;N_{48}$, and $N_{51}$ in the school and $N_{37},\;N_{41},\;N_{46},\;N_{52},\;N_{61}$, and $N_{69}$ in the hospital) were identified to ensure that the shooter was moving throughout the entire building and providing a variety of situations in which the evacuees would have to react. Accounting for all of the parameter combinations, the school and hospital were used to examine 3,456 and 9,028 different cases respectively.

## 5 Results

### 5.1 Overall performance

**Figure 4:**
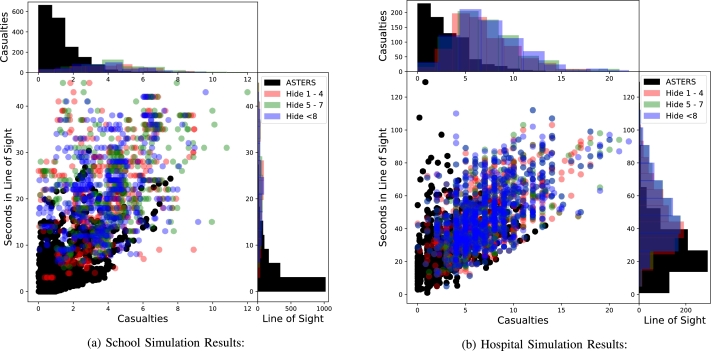
Overall simulation results for school and hospital: each dot is color coded to represent the optimized algorithm and the naturalistic logic. The optimized routing algorithm data in each of the histograms are densely scattered around 0 casualties and 0 seconds spent in line of sight of the shooter. The distributions for casualties and LOS are separately seen along the two axes.

The optimized routing algorithm was compared to each of the Naturalistic Response plans (corresponding to the different proximity thresholds ranging from a path-length of 1 up to 8) on both the single-level school and the two-level hospital layouts. The two graphs depicting all of the simulation results combined for the school and the hospital are in Fig. 4a and 4b.

The horizontal axes in these graphs are the number of casualties and the vertical axes are the time spent within the shooter's line of sight. As the legend in the figure suggests, the NR algorithms are parameterized by the proximity threshold and are grouped because they either performed exactly the same or with negligible difference. These graphs show the average of 50 runs in each of the 3,456 scenarios in the school and 9,028 scenarios in the hospital based on the combination of factors discussed earlier. The density histograms show that the concentration of the optimized algorithm's performance is significantly closer to 0 casualties as well as minimum time spent in the shooter's line-of-sight while the *NR* algorithms tend to be much more spread along the respective axes towards higher casualties and higher exposure to danger.

The school, which is much less complex architecturally than the hospital, shows a very clear advantage to the optimized performance in the sense that both casualties and LOS time are minimized. The more complex hospital demonstrates a more interesting phenomenon in the sense that even though casualty is minimized for our optimized plan, it does lead to isolated cases of evacuees getting long exposure in the line of site of the shooter. This discrepancy actually highlights the power of the optimized algorithm since it can distinguish between the fact that spending more time far away from the shooter even though in the line of sight can provide viable escape paths with low chance of death and actually urges the evacuees to utilize these paths. In real life, such fine tuned escapes may not be possible due to the uncertainty and delay in execution of the proposed plan, but this exercise does provide a successful algorithmic baseline which may be fine tuned based on human factor considerations.

While these graphs give a general idea of the performance, more quantitative measures can be observed from Table 3 which categorizes the tested simulated conditions to demonstrate different performance levels based upon the particular common parameter conditions present. For each set of conditions, the simulation was run 50 times for each of the 9 algorithms – (optimized and $NR_{1}$ through $NR_{8}$) The values reported in the table are the averages of the 50 runs. In this table, for clarity we only compared the performance of the optimized algorithm to the best performing of the eight NR algorithms in each parameter scenario.

**Table 3:** Tabular Simulation Results optimized VS the best NR responses: This table shows a pairwise comparison of the optimized algorithm with the best performing NR for each combination of parameters for both the school and hospital assets. The ASTERS routing algorithm outperforms the best NR in each and every parameter combination across both assets in both casualties and time spent in line of sight.

Shooter spawn	Evacuee distribution	Evacuee speed	Hospital				School			
			ASTERS		Best NR		ASTERS		Best NR	
			Casualty	LoS	Casualty	LoS	Casualty	LoS	Casualty	LoS
Exit	Rooms	0.5	2.7	20.8	5.6	34.2	0.7	4.2	2.6	18.4
		0.75	1.7	16.2	5.4	33.7	0.3	2.8	2.9	17.7
		1.0	0.6	11.8	5.7	34.0	0.3	1.3	3.0	18.1
									
	Rooms and Halls	0.5	4.4	35.6	8.8	53.2	1.0	10.1	4.4	28.8
		0.75	2.6	35.6	8.8	53.2	1.0	10.1	4.4	28.8
		1.0	1.1	18.6	8.1	52.1	0.6	5.8	5.1	28.3

Hall	Rooms	0.5	2.7	20.6	5.0	33.9	1.0	3.8	1.5	14.5
		0.75	1.1	15.7	5.0	35.7	0.7	1.9	1.3	15.5
		1.0	0.8	11.3	4.5	35.7	0.4	1.4	1.0	14.5
									
	Rooms and Halls	0.5	5.7	39.1	8.6	58.8	2.6	12.2	3.4	25.6
		0.75	3.0	30.1	7.9	61.0	1.9	9.4	3.2	27.3
		1.0	2.3	22.5	7.7	60.4	1.6	8.0	2.6	26.2

Room	Rooms	0.5	3.9	21.9	6.3	33.7	2.0	5.9	2.5	18.9
		0.75	2.2	16.0	5.8	34.1	1.3	2.2	3.1	15.8
		1.0	1.9	10.8	4.8	34.4	1.2	1.5	2.7	16.0
									
	Rooms and Halls	0.5	6.3	37.9	9.6	52.1	2.6	8.8	3.3	25.5
		0.75	3.7	27.9	8.3	53.0	1.4	3.4	4.3	21.1
		1.0	3.0	21.3	7.0	52.8	1.3	2.3	3.7	21.3

Stair	Rooms	0.5	3.8	30.0	5.1	38.5				
		0.75	2.8	28.1	4.4	36.3				
		1.0	2.6	24.8	4.3	36.4				
									
	Rooms and Halls	0.5	5.3	44.7	8.0	58.6				
		0.75	3.6	36.8	7.0	55.6				
		1.0	3.2	33.9	6.4	55.7				

Table 3 summarizes the results – the optimized algorithm outperformed the best NR algorithm in each of the parameter scenarios in both the school and the hospital. Across all combinations of parameters, following the optimized algorithm resulted in 56% fewer deaths and 52% less time spent in the shooter's line of sight in aggregate. The averaging over 50 iterations with randomly varying movement pattern for the shooter enforces robustness of the results produced. Moreover, since the random generator is seeded identically, the exact same (random) conditions are tested for all the algorithms, thus guaranteeing the consistency across the tests. Going beyond the gross statistics, we also examined our algorithm's performance individually across the different scenarios.

### 5.2 Shooter's start location

The location where the shooter was first spotted played an interesting role in the effectiveness of the algorithms' ability to route individuals to safety. There were nine shooter start locations for the school to provide a variety of situations for the evacuees to respond to. For the simulation, we assumed the spawning of the shooter to be initialization of the active shooter event. The school shooter spawned in three hallway nodes (3, 6, and 16), two room nodes (26 and 48), the cafeteria (51), and three of the four exits (52, 53, and 54). The hospital shooter spawned in six hallway nodes (2, 9, 15, 18, 21, 25, and 30), six room nodes (37, 41, 46, 52, 61, and 69), three stairway nodes (72, 74, and 77), and all of the exits (78, 79, 80, 81, and 82). These spawn locations for both the school and hospital were identified to ensure a variety of situations were examined.

**Figure 5:**
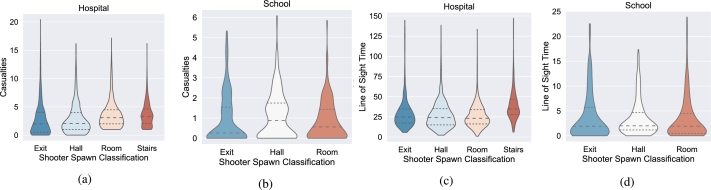
Overall The violin plots for casualties and time spent in line of sight distinguished by Shooter Spawn Location: a) Spawning the shooter inside rooms in the hospital incur increased casualties as a number of evacuees immediately get caught by the shooter. Stair spawns allow the shooter to move to either floor making evacuation decisions difficult; both result in higher mean casualties across all runs, b) the simpler school asset presents fewer layout challenges, but the hallway spawns incurred a higher mean casualty rate as well as more occurrences of higher casualties, c) the stairways in the hospital provide a significant disadvantage due the uncertainty of which floor the shooter will be on increasing the mean and upper bounds of the time spent in line of sight, and d) again, the simpler layout of the school did not demonstrate significant differences in the time spent in line of sight which really highlights the affect the stairways had.

As it can be seen in Fig. 5c and 5d, the time in line of sight stays fairly constant across the board regardless of where the shooter spawns. An encouraging result of the simulation is the number of deaths in both buildings is decreased by at least 25% when the shooter spawns in an exit node (this means the shooter is identified before they enter the building). The shooter spawning in Hall and Room were far more deadly for the evacuees, but not significantly different from each other. The hospital, in particular, showed similar trends based upon the shooter spawn locations, however, since it is a more complex building with longer hallways and sight-lines, multiple floors, and more nodes, the contrast in performance based upon the spawn locations was not as evident.

What does this physically mean in the real world? When we are able to identify a shooter before they enter the building, we are more often able to direct evacuees to safety. However, when the shooter cannot be identified until they are in a room or hall, the deaths that occurred were always greater than or equal to 1 indicating that it may be too late to save everyone if the shooter is already in the building. When planning for security purposes, it may be necessary to apply more resources to the visual and physical identification of the shooter before they enter a building. These resources could take the form of a school resource officer, metal detector, or, as we are assuming for the purpose of this paper, computer vision assisted cameras.

### 5.3 Evacuee distribution effects

**Figure 6:**
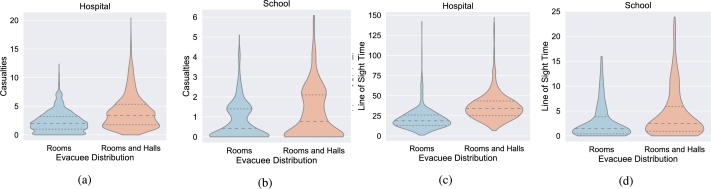
Violin plots for casualties and time spent in line of sight for both the hospital and school partitioned by the distribution of the evacuees. While the shape distributions of the hospital and school casualties are different, they each share the common aspect that evacuees distributed in the rooms only at the start of an active shooter incident incurred fewer mean casualties and lower ceilings of maximum casualties. Similar trend was observed for the LOS as well.

For both the school and the hospital, we tested two evacuee distributions: uniformly distributed only in the rooms and in both rooms and halls. As discussed earlier, the distributions had different meanings for each of the building layouts, but, in essence, are the same in the way they are implemented. Both the optimized algorithm and the best NR performed better when only the rooms were occupied at event initiation as seen in Fig. 6a and 6b resulting in a 40% reduction in casualties across both buildings for the optimized plan and 43% reduction for the best NR. Evacuees present in both rooms and halls led to increased deaths in both buildings and also resulted in the some of the worst performances for the optimized algorithm among all the parameter combinations.

Thinking of this from a policy perspective, it is of course not practical to prevent people from using hallways in order to deter a shooter in one of these situations. That being said, there are potentially directives that can at least reduce the number of people in hallways and therefore increase their ability to maximize safety. An interesting idea was used on several college campuses during the 2020–2021 academic year to try and socially distance their students as much as possible. These universities staggered class times throughout the day in order to prevent the mass movement of students through hallways and walkways when all classes begin and end at the same time. By staggering the class ending times, they managed to reduce the number of people moving around on campus at any given point. A similar structure could work inside of a building as well to reduce the number of people in a hallway and at the same time avoiding the significantly slower moving times that occur when every class ends at the same time.

### 5.4 Evacuee speed

The movement speed of the evacuees, both in the real world and the simulation, plays an enormous role in the performance the algorithm. There are vast differences between the movement speed of an adult and that of a group of young children. Further, there is a difference in the movement speed of someone who is calm, in no hurry, and someone is frantic in a dangerous situation. The simulation allowed the evacuees to homogeneously have different movement speed, meaning that all of the evacuees had the same speed, but it changed in conjunction with specific parameter combinations. The speeds the evacuees were able to move at were related to the speed of the shooter. A movement speed of 0.75 indicated that they moved at 75% of the shooter's speed.

**Figure 7:**
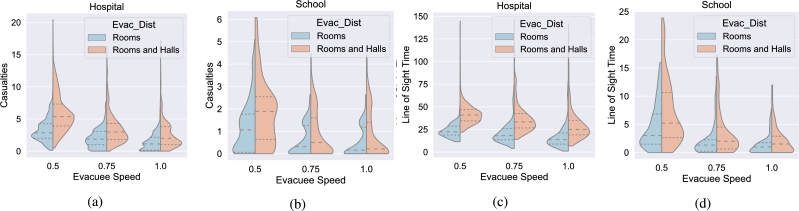
Violin plots for casualties and time spent in line of sight for both the hospital and school partitioned by the evacuees' movement speed and also by evacuee distribution. There are two common themes in every chart: 1) the slower the evacuees' speed was, the larger the mean casualties and mean time spent in line of sight and 2) considering each violin as a pairwise comparison within each evacuee speed, the room only distribution has fewer mean casualties and spends less time in line of sight in every pairing.

The algorithm used the same speed as the shooter as a planning factor for the evacuees routing. So, evacuees with less speed than the shooter still used the routing meant for faster evacuees. The results of these speed differences can be seen in Fig. 7. The deaths and time in line of sight can be seen in the violin plots, further broken down by evacuee distribution and building type. As it can be seen in the graphs, in each of the different scenarios between the hospital, school, and evacuee distributions, as the evacuees' speed increased, the amount of time they spent in the shooter's line-of-sight decreased making their ability to stay safe easier. The downward trend was the same for deaths in each of the scenarios as well. The difference in deaths and time in line of sight between the fastest and slowest movement speeds was 53% and 41% respectively, which is a significant difference. Looking forward, taking the speed of an evacuee into account and developing a plan specific to each group can only provide better results in the future.

### 5.5 Cameras and position updates

As discussed earlier, this algorithm is assumed to complement a camera-based shooter-identification and tracking system. It is quite evident that the algorithm's effectiveness in providing optimal and timely guidance to the evacuees will depend on the frequency and quality of the updates it receives from the monitoring system. In order to investigate the routing algorithm's dependence on the performance of the camera network, we next look at a related problem – if resources are limited, and full coverage of the entire building with a dense network of cameras is not possible, what would be the optimal locations to place cameras so that the performance of the routing algorithm is still maximally preserved.

To solve the camera placement problem, we used the characteristics of the graph networks which were derived from the floor plans of the building we were instrumenting. The goal was to logically add the cameras to the model without redundant line of sight. We used the network adjacency matrix and calculated the betweenness centrality, a measure of how many shortest paths the node appears in, of every node. The choice of the betweenness centrality as the metric for camera placement is related to the underlying unbiased movement model adopted for the shooter's random walk. Such random walk would result in an eventual steady state probability distribution of the shooter's possible locations over the nodes of the graph. This distribution again is related to the “connectivity” of a node, i.e. the more number of ways a node is accessible, the large is the chance of the shooter to pass through that node. This in turn makes it a logical choice for placing a camera.

Once the betweenness centrality is calculated for the entire building graph, we then rank-ordered the nodes from greatest to least betweenness centrality measure and reduced the list by removing all of the nodes that had a measure of 0, meaning that the shortest paths they appeared in were the ones either beginning or ending with themselves. We then iteratively added a camera to the node with the largest betweenness centrality and then removed any node from the ordered list that was in the added camera's line of sight until the list was empty. Each of the camera locations for the school and hospital will be covered in the following sections.

#### 5.5.1 Camera performance for school

**Figure 8:**
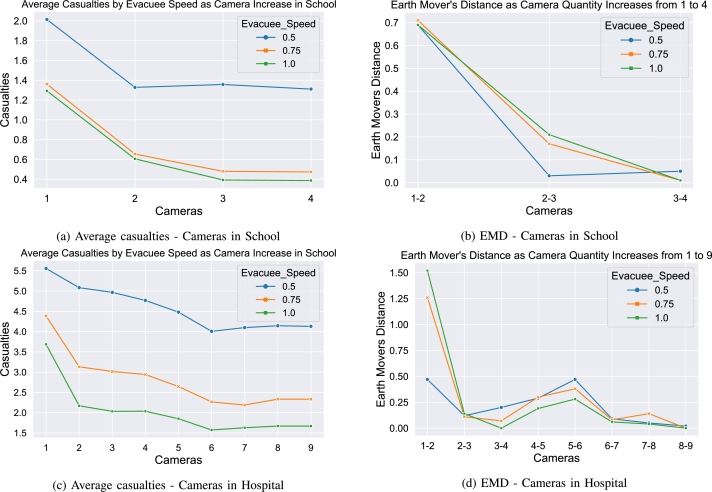
Affect of cameras in the school and hospital building layouts: *a:* The average casualties decrease as more cameras are added to the schools hallways indicating that increased awareness of the shooter's location provides increased safety protocols for the evacuees. *b:* The earth mover's distance (EMD) shows the amount of change when increasing the number of cameras, thus the large decrease in average casualties when increasing cameras from one to two, but much smaller decreases when increasing cameras from there. *c:* The average casualties steadily declines as more cameras are introduced, and potentially hitting a saturation point where more cameras provide negligible benefit at 7 cameras. *d:* while the EMD from one to two cameras is large, the addition of cameras after the second shows a little benefit, but not as drastic as one to two, which is explained by the two floor layout of the hospital and increasing to two cameras puts one on each floor. Beyond the 7^*th*^ camera, the effect of adding additional cameras almost falls to 0.

Using the methods previously discussed, we established four possible locations for the cameras in the school: *Nodes* $N_{6},N_{8},N_{14}$*, and* $N_{51}$, in that order. For each set of parameters, a camera was added iteratively and run 50 times. The results of iteratively adding the cameras into the simulation demonstrate the increasing effectiveness of the optimized algorithm as the amount of information on the shooter's position increases. Fig. 8a and 8b show the output from these runs. The average deaths, broken down by evacuee speed, show the decrease in the deaths as the number of cameras in the simulation increase. On a side note, it also demonstrates the effect the difference in evacuee speeds has on the performance as well. The other figure shows the Earth Mover's Distance (EMD) for the distributions of casualties with addition of cameras. EMD is a distance measurement that quantifies how different two distributions are with values close to 0 being extremely similar and larger values being more dissimilar. As it can be seen, there is a significant difference between one and two cameras, a little less than half of the difference between two and three cameras, and almost no difference adding the fourth camera. Both the average deaths and the EMD mirror the same conclusions: performance continues to improve as cameras are added to the simulation providing more and more regular updates to the shooter's position. However, when having to take into account possible budgetary concerns for schools, the fourth camera in *Node 51*, the cafeteria, does not provide a significant jump in performance and could be potentially removed in the name of cost savings.

#### 5.5.2 Camera performance for hospital

The hospital, due to its more complex structure and quantity of nodes, had nine cameras in the following locations: *Nodes* $N_{22},\;N_{6},\;N_{13},\;N_{76},\;N_{28},\;N_{71},\;N_{73},\;N_{62}$*, and* $N_{50}$, in that order. The performance for the algorithms as the cameras are added iteratively can be seen in Fig. 8c and 8d. The average casualties in the hospital steadily drop as the cameras are added providing the additional information on the shooter's position. Again, it also demonstrates the vast difference that evacuee speed plays in the performance of the algorithm. Deaths significantly drop when adding the second camera and then steadily decline when adding cameras 3, 4, 5, and 6. However, much like with camera 4 in the school, adding additional cameras provides significantly less benefit past camera 6 (diminishing returns). The EMD for the hospital camera distributions demonstrates the same trend with a big difference between one and two cameras, and very little difference in the changes between cameras three and six. The additional cameras past six again show very little difference in the distributions and hence very little benefit to the performance.

## 6 Conclusion and future work

In this work we have shown that the route planning in the event of an active shooter situation can be achieved using a naive NHSMDP approach. The reward structure that balances the benefit of being in a safer location against the benefit of moving closer to an exit while taking into account the location of the shooter allows the optimized routing algorithm to identify when it is safer to wait in a room or begin moving to an exit which is unique across all the other route planning algorithms. Across all combinations of parameters that we examined, the optimized routing algorithm outperformed the Natural Response plans by 56% fewer casualties and 52% less time spent in the shooter's line of sight in aggregate.

It is evident that the process of egress and optimization represented here is an abstraction of the true situation. Even within the bounds of our simplifying assumptions, there are several improvements that will potentially make this method a practical viable safety tool. Some of the current limitations of this work comes from lack of

• consideration of psychology, human factors and heterogeneity among the evacuating group,
• consideration of capacity and bottle-necking at doors and corridors, and
• ethical considerations

The most important limitation of this study is in the treatment of the evacuees as a perfectly compliant, logical, efficient homogeneous group of people. Obviously, none of these attributes are true in general, and especially for people under stress. Literature shows that human factors, such as social pressure, leader follower, route familiarity and influence from trusted sources all play significant parts in the evacuees' choices and behavior [15], [27], [28]. Knowing the stress and danger involved in an active shooter situation, we want to further develop the algorithm to account for hesitancy, or even refusal, of the evacuees to obey the routing plans. Multiple factors will play into that work to include fear of the danger posed, trust/mistrust of a computer directed routing plan, etc. Heterogeneous populations with different abilities and capabilities will need to be accounted for to increase the applicability of the algorithm across multiple scenarios and complexities. Routing people of different ages and capacities, modeled as different speeds and instruction following accuracy, at the same time with different speed optimized routes will provide better feedback on how we can best assist schools with widely varying ages in these situations.

The capacity of a node or an edge is also an extremely important factor, especially for larger buildings like schools, office buildings, hospitals, etc. where a large number of people may spill into a hallway at the same time causing congestion. The algorithm should be able to account for that situation, develop plans for everyone in the building based upon available capacity, and direct some evacuees to hide in their current node for a period of time in order to prevent bottle-necking in the hallways and exits, where people are most vulnerable due to crowding and lack of cover.

In its current abstracted form, the merit of this work is in its effectiveness in developing an algorithm that successfully guides evacuees to safety in response to a mobile threat. Building upon this framework, the algorithm will be augmented in the future with more realistic considerations. However, one of the most difficult challenges in the realization of such algorithmic solutions is not technical, but ethical. We realized that significant moral dilemmas will need to be overcome when prioritizing evacuation routes of some groups over others, when capacity constraints demand such coordination. The amount of tolerable risk posed by the shooter and balancing that with the chance of escaping needs to be carefully considered. Taking into account these issues will continue to improve our performance as well as add realistic dimensions to the routing plan. However, similar to the famous trolley problem, the ability to make such nuanced decisions beforehand and codifying them in an algorithmic logic will no doubt need discussions with experts in various fields of psychology, education and planning. It will not be possible to reach fair and equitable algorithmic solutions without some difficult moral and ethical debates.

## Funding statement

Dr. Subhadeep Chakraborty was supported by the National Science Foundation 10.13039/100000083Directorate for Computer and Information Science and Engineering [1932505].

## CRediT authorship contribution statement

Joseph Lavalle-Rivera: Conceived and designed the experiments; Performed the experiments; Analyzed and interpreted the data; Wrote the paper. Aniirudh Ramesh, Subhadeep Chakraborty: Conceived and designed the experiments; Analyzed and interpreted the data; Wrote the paper. Laura M. Harris: Performed the experiments.

## Declaration of Competing Interest

The authors declare no competing interests.

## Data Availability

Data will be made available on request.
